# Women in Neuromodulation: Innovative Contributions to Stereotactic and Functional Neurosurgery

**DOI:** 10.3389/fnhum.2021.756039

**Published:** 2022-01-20

**Authors:** Petra Heiden, Julia Pieczewski, Pablo Andrade

**Affiliations:** ^1^Department of Neurosurgery, Faculty of Medicine, University of Cologne, Cologne, Germany; ^2^Department of Stereotactic and Functional Neurosurgery, Faculty of Medicine, University of Cologne, Cologne, Germany

**Keywords:** functional neurosurgery, neuromodulation, stereotactic surgery, deep brain stimulation, history, women

## Abstract

Stereotactic neurosurgery emerged in the mid-20th century following the development of a stereotactic frame by Spiegel and Wycis. Historically women were underrepresented in clinical and academic neurosurgery. There is still a significant deficit of female scientists in this field. This article aims to demonstrate the career and scientific work of some of the most important women who contributed to the development of stereotactic and functional neurosurgery. Exceptional women from all over the world, represented in this review, assisted the evolution of modern stereotactic and functional neurosurgery as neurosurgeons, neuropathologists, neurologists, neurophysiologists and occupational therapists. Fortunately, we could conclude that in the last two decades the number of female researchers has increased significantly.

## Introduction

Stereotactic and functional and neurosurgery emerged towards the mid-20th century when Spiegel and Wycis first designed a stereotactic frame for use on the human brain (Spiegel et al., 1947). This frame was based on the apparatus developed for animal experiments by Horsley and Clarke (1908). The rationale to develop a human frame was to allow the creation of circumscribed lesions in deep brain structures in psychiatric patients in order to avoid the severe complications seen after the crude frontal lobotomies (Gildenberg, 2004; Rzesnitzek et al., 2019). This method initially labeled stereoencephalotomy and subsequently stereotactic surgery, was eventually implemented also in the treatment of patients with chronic pain, movement disorders, and epilepsy.

Historically and until the 1990s, female scientists were very rare in the fields of neurosurgery and functional neurosurgery, including neuromodulation, and they are still underrepresented in higher academic positions in these fields (Renfrow et al., 2018; Schaller, 2020). Several factors were detected contributing to gender inequality in neurosurgery (Venes and Parent, 2006; Abosch and Rutka, 2018). Women are still commonly identified as primary caretakers of the family; thus, it is difficult to adjust inflexible working hours of neurosurgical training and academic work environment to pregnancy and childcare. Women might also be discouraged by the lack of female leaders and role models in this field. Reports also have shown that women are less likely to have protected research time, an office or laboratory space, or receiving grant support (Venes and Parent, 2006; Abosch and Rutka, 2018). There are, however, few women who did contribute to the evolution of stereotactic and functional neurosurgery (Hariz et al., 2014). The aim of this article is to acknowledge and report the innovative work of female scientists to the development of this field.

### Movement Disorders

#### Marion Smith (1915*–1988†), Neuropathologist

**Figure 1:**
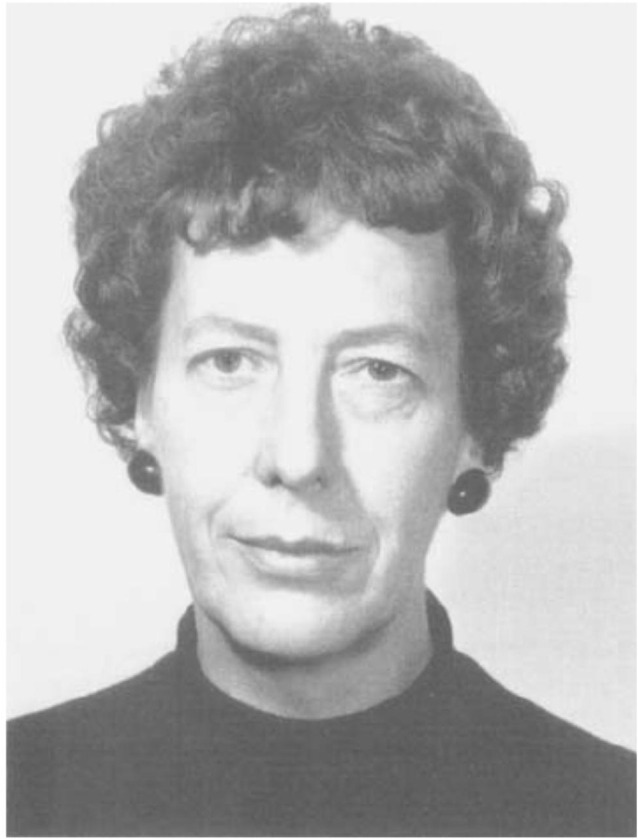
Marion Smith, reproduced from Duchen (1989).

Marion Smith (Figure 1) was a neuropathologist and neuroanatomist born in Glasgow in 1915. She graduated in zoology before completing her medical studies at the Western Infirmary in Glasgow. She was trained in the field of neuropathology by Dr. Joseph G. Greenfield at the National Hospital for Nervous Diseases at Queen Square in London, where she continued to work until her retirement at the age of 65. She was a founding member and later president of the British Neuropathological Society (Duchen, 1989).

Her research contributed greatly to the anatomical and functional understanding of the brain and the spinal cord (Nathan and Smith, 1955, 1982; Smith and Deacon, 1984; Nathan et al., 1996). Her studies on post mortem brains of patients who had stereotactic lesioning provided a great insight into the effects and side-effects of the surgery. One of her most important contributions—in an era where there was no brain imaging available—was an analysis of thalamotomies and pallidotomies in 15 patients with Parkinson’s disease, hemichorea, or Huntington’s disease (Smith, 1962). She described in detail the location and size of the lesions and the involvement of different anatomical structures, and their correlation with the clinical outcome of the patients.

The clinicopathological correlations derived from her post mortem studies provided greatly to optimizing targets for future stereotactic surgeries.

#### Natalia Petrovna Bechtereva (1924*–2008†), Neurosurgeon

**Figure 2:**
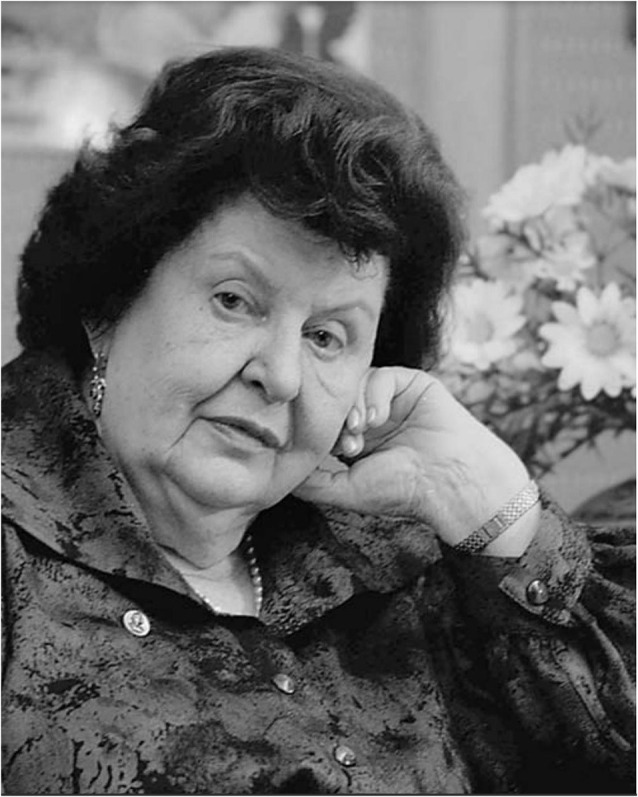
Natalia Bechtereva, reproduced from Anonymus (2008).

Natalia Bechtereva (Figure 2) was a neurophysiologist and neuroscientist born in Leningrad in 1924. She graduated from the Pavlov Institute of Physiology after graduating from the Pavlov First Leningrad Medical Institute and started her research career at the Polenov Institute of Neurosurgery in Leningrad in 1954 (Anonymus, 2008).

From the very beginning of stereotactic surgery, Spiegel and Wycis relied on intraoperative electrical stimulation in order to confirm the correct location of the electrode before performing lesioning (Gildenberg, 2005). Intraoperative acute stimulation continued to be part of the lesional procedures in the following decades (Blomstedt and Hariz, 2010). Natalia Bechtereva was the first to introduce a method for chronic electrical stimulation as a therapy for movement disorders and chronic pain (Blomstedt and Hariz, 2010; Hariz et al., 2014). Starting in the 1960s, she implanted gold electrodes for chronic external stimulation of different subcortical targets, including the ventrolateral thalamus and the striatopallidal complex, in some cases targeting multiple structures in the same patient (Bechtereva et al., 1975). She called her method therapeutic electrical stimulation (TES), and she used a current with “high-rate pulses” (Bechtereva et al., 1975), meaning a stimulation with high frequency. The electrodes remained implanted for up to 1.5 years with stimulation sessions conducted once or twice a week through an external stimulator. Her reports showed a significant improvement of the symptoms with few relevant side effects (Bechtereva et al., 1975).

As her first publications were in Russian, her work was not well known in the Western scientific community until her first publications in English appeared in 1975 (Blomstedt and Hariz, 2010; Hariz et al., 2014).

#### Thanjavur Santhanakrishna Kanaka (1932*–2018), Neurosurgeon

**Figure 3:**
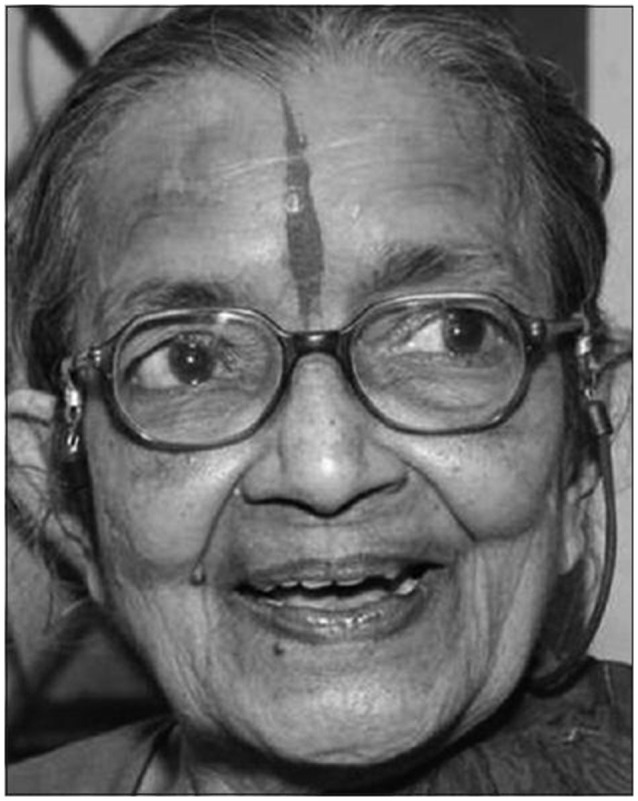
Thanjavur Santhanakakrishna Kanaka, reproduced from Vilanilam et al. (2016).

Thanjavur Santhanakakrishna Kanaka (Figure 3) was a neurosurgeon born in Chennai, India in 1932. She finished her medical studies in 1954 at the Madras Medical College where she continued specializing in neurosurgery and became the first female neurosurgeon of India in 1968 (Ganapathy and Barreto, 2021).

Under the guidance of Prof. B Ramamurthi she gained her Ph.D. degree researching stereotactic surgery for cerebral palsy and later joined the Madras Institute of Neurology which soon became the center of stereotactic surgery in India (Ozair et al., 2021). Within 15 years Dr. Kanaka and her colleagues performed more than 1,700 surgeries treating involuntary movements, behavioral disorders, psychiatric disorders, epilepsy, and spasticity (Ramesh et al., 2015). She also covered many pathologies in her work as a researcher. She studied thalamotomies and dentatectomies as well as the combination of different targets in patients with cerebral palsy, establishing the basis for symptom-oriented individualized treatment of the patients depending on their symptoms (Balasubramaniam et al., 1974). She also performed studies researching cingulotomy in drug addiction and hypothalamotomy in hyperkinetic behavioral disorders (Balasubramaniam et al., 1973; Kanaka and Balasubramaniam, 1978). Dr. Kanaka was also the first neurosurgeon who performed chronic deep brain stimulation in India (Ozair et al., 2021).

Dr. Kanaka faced great difficulties starting her carrier as a female surgeon in India. As a result of systematic discrimination against women, she required a number of attempts even to qualify for a residency program. During her residency, she was denied training by the chief resident and it also took several tries for her to pass the exit exam. To support female neurosurgeons across Asia she founded the Asian Women’s Neurosurgical Association in 1996 together with Yoko Kato, the first professional female neurosurgeon in Japan (Ozair et al., 2021).

#### Hilda Molina, Neurosurgeon

**Figure 4:**
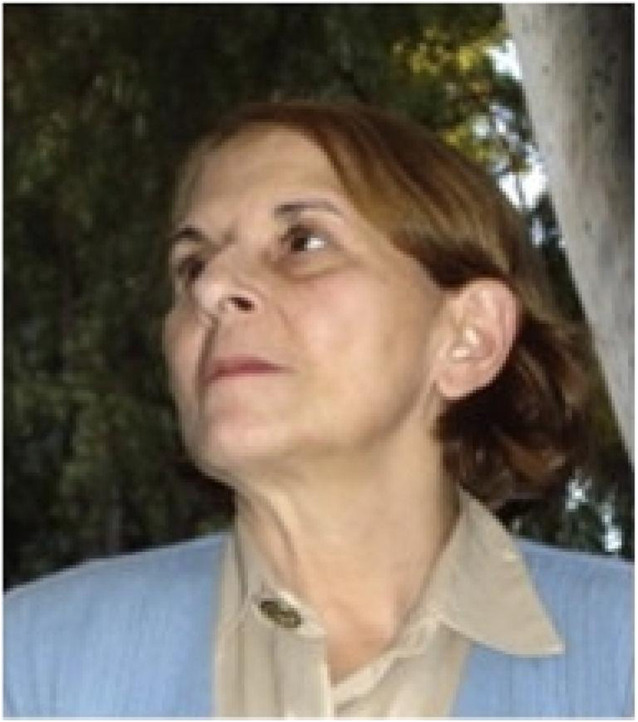
Hilda Molina, reproduced from Hariz et al. (2014).

Hilda Molina (Figure 4) was a neurosurgeon born in Cuba in 1943. She graduated from La Havana University in 1974, then specialized in neurosurgery, finishing her training in 1978 as the first female neurosurgeon in Cuba.

She later gained a Ph.D. degree studying multiple saccular aneurysms. She was founder of the Centro Internacional de Restauración Neurológica (CIREN) in Cuba, where she researched neurotransplantation in patients with Parkinson’s disease. Studies performed in the 1980s on 1methyl-4-phenyl-1,2,3,6-tetrahydro-pyridine (MPTP) non-human primate models of Parkinson’s disease, showed promising results after their treatment with fetal neural grafts (Redmond et al., 1986; Bankiewicz et al., 1990). In 1987, Molina and her colleagues implanted fetal mesencephalic tissue into the caudate nucleus using open surgery techniques (Molina et al., 1992). In 1992, they performed the first CT-guided stereotactic transplantation, implanting neural graft in the caudate nucleus and the putamen (Molina et al., 1994). They even used microelectrode recordings to confirm the right implantation location (Molina et al., 1994). Hilda Molina performed stereotactic thalamotomies in the nucleus ventralis intermedius also in patients who previously received neural grafts but continued to suffer from severe tremors (Quiñones-Molina et al., 1994). Dr. Molina retired in 1994 and lives now in Buenos Aires, Argentina.

#### Gun-Marie Hariz, Occupational Therapist

**Figure 5:**
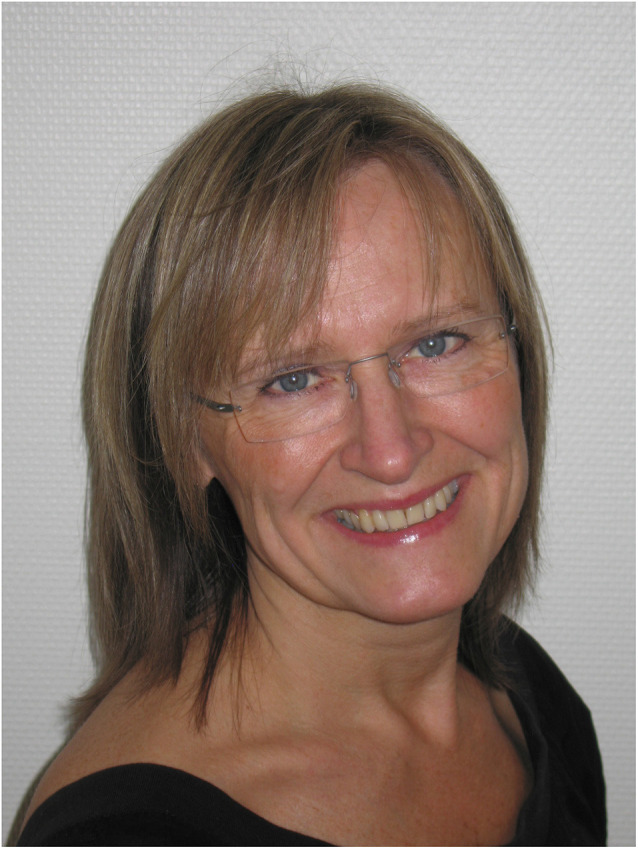
Gun-Marie Hariz, provided by Gun-Marie Hariz.

Gun-Marie Hariz (Figure 5) is an occupational therapist and researcher born in 1954 in Sweden. She obtained her certification in Stockholm in 1979. In 1999 she received a Master of Science and in 2002 a Ph.D. degree at Umeå University where she continued her work as a researcher.

Early on in her research career, she focused on the quality of life of patients with movement disorders, with particular interest in patients treated with DBS (Hariz et al., 1998). Being an occupational therapist, Gun-Marie Hariz has been interested in the concrete challenges met by patients in their activities of daily living, as reported by them. In this respect, she focused on patients’ perception of their disease and its treatment (Hariz et al., 2011). For this, she has thoroughly documented the importance of applying qualitative investigations to illustrate the real difficulties met by the patients instead of using solely quantitative methods and scales. The insights obtained from her research have allowed clinicians to remain critical about the use of standardized scales as a unique tool to determine a positive clinical outcome. In addition, she has led the field on investigating social and individual aspects in patients with DBS that have traditionally been underestimated or ignored since the introduction of this therapy. Here, she has focused particularly on gender inequalities in access for persons with Parkinson’s disease to surgical treatment (Georgiev et al., 2017; Sperens et al., 2017). Likewise, her research has opened the discussion on topics that are unfamiliar to traditional clinical research, although extremely relevant for patients, such as the perception of living with implanted devices and the experience of managing these systems (Hariz and Hamberg, 2014). These deep analyses of the perception of these patients provide powerful insights into the reality and impact of living with DBS, which should be considered by physicians and researchers at every moment of the treatment. In 2012, she was appointed Associate Professor of Occupational Therapy at the Department of Community Medicine and Rehabilitation at the Umeå University, and a senior researcher at the Department of Clinical Neuroscience, where she currently holds this position.

#### Patricia Limousin, Neurologist

**Figure 6:**
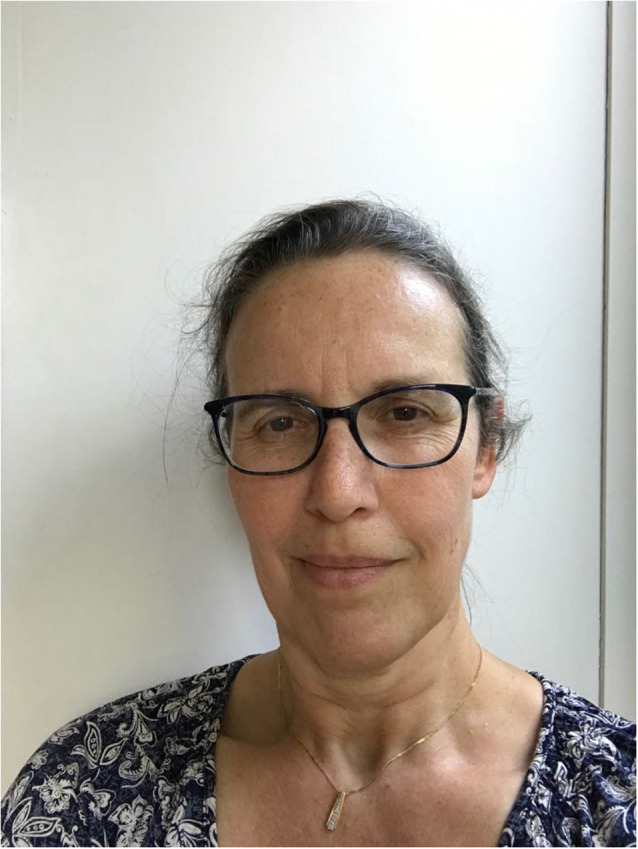
Patricia Limousin, provided by Patricia Limousin.

Patricia Limousin (Figure 6) is a neurologist born in 1965. She received a Master of Science degree in 1991 and after completing her specialization in neurology in 1993 at the University of Grenoble she joined the research team of Benabid and Pollak.

In the late 1980s, Alim Louis Benabid and Pierre Pollak in Grenoble, France, initiated the modern era of deep brain stimulation (DBS) using eventually a fully implantable system (Benabid et al., 1987). They started by targeting the ventro-intermediate (VIM) nucleus of the thalamus for patients with Parkinsonian or essential tremor (Benabid et al., 1987, 1991), showing a very good effect on the tremor. However, VIM DBS did not provide relief from other symptoms of advanced Parkinson’s disease, such as bradykinesia, dyskinesias, and gait abnormalities (Limousin and Martinez-Torres, 2008).

Based on studies of the role of the subthalamic nucleus (STN) in animal models of Parkinson’s disease (Bergman et al., 1990), Dr. Limousin and her colleagues introduced the STN as a new DBS target in Parkinson’s disease showing in a seminal article on bilateral DBS of the STN, published in The Lancet in 1995, that this procedure also improved bradykinesia and rigor significantly (Limousin et al., 1995). In the following years, STN DBS became the most common target used worldwide for DBS in Parkinson’s disease (Limousin and Martinez-Torres, 2008).

Dr. Limousin proceeded to obtain her Ph.D. in neuroscience at the University of Lyon, continuing her pioneering work on the study of STN DBS. In the late 1990s, she moved to work at the Institute of Neurology at Queen Square in London where she eventually contributed to the establishment of a Unit of Functional Neurosurgery. Currently, she is a Professor of Clinical Neurology and Consultant Neurologist at the National Hospital for Neurology and Neurosurgery and at University College London.

#### Carine Karachi, Neurosurgeon

**Figure 7:**
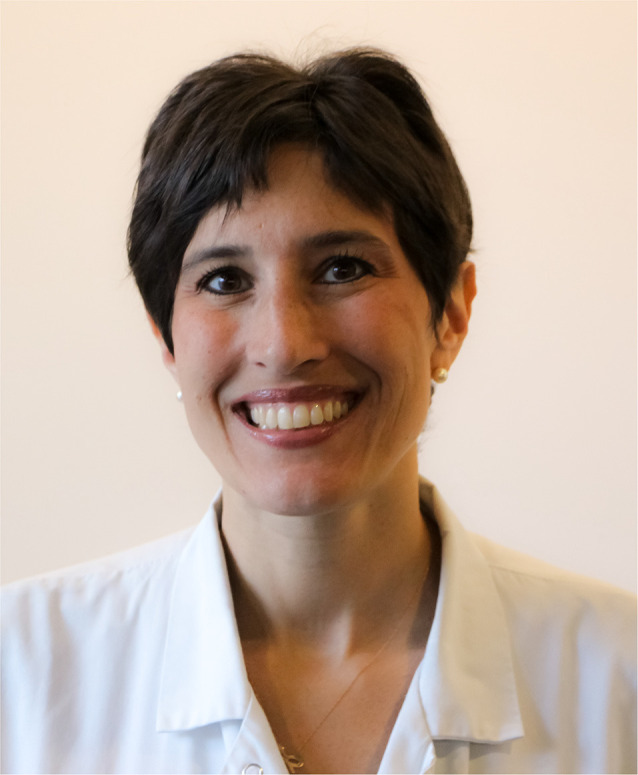
Carine Karachi, provided by Carine Karachi.

Carine Karachi (Figure 7) is a French neurosurgeon and neuroscientist born in 1973. After finishing her medical training in Paris, she defended a Ph.D. thesis on the function and anatomy of the basal ganglia in 2006. She incorporated the insights obtained during laboratory research into the clinical field. This is exemplified by her composite formation, which includes a postdoctoral fellowship in basic sciences at Columbia University in New York and neurosurgical training at the Pitié-Salpêtrière Hospital in Paris.

Her main focus in the field of DBS is on the pathophysiology of gait and balance disorders in patients with movement disorders, in particular on the role of the pedunculopontine nucleus (PPN) and its interaction with other network structures involved in locomotion. Her extensive contribution in this area has increased our understanding of the complex role of cholinergic neurons located in this part of the brainstem and their involvement in posture and locomotion in Parkinson’s disease (Karachi et al., 2010; Grabli et al., 2013). Moreover, these insights have contributed to the treatment of these disturbances with DBS of the PPN. Currently, Carine Karachi (who was nominated Professor in 2019) combines her dual expertise working as a neurosurgeon at the Pitié-Salpêtrière Hospital and as a lead researcher at the Institut du Cerveau et de la Moelle Èpinière (Brain and Spine Institute) in Paris.

#### Laura Cif, Neurologist

**Figure 8:**
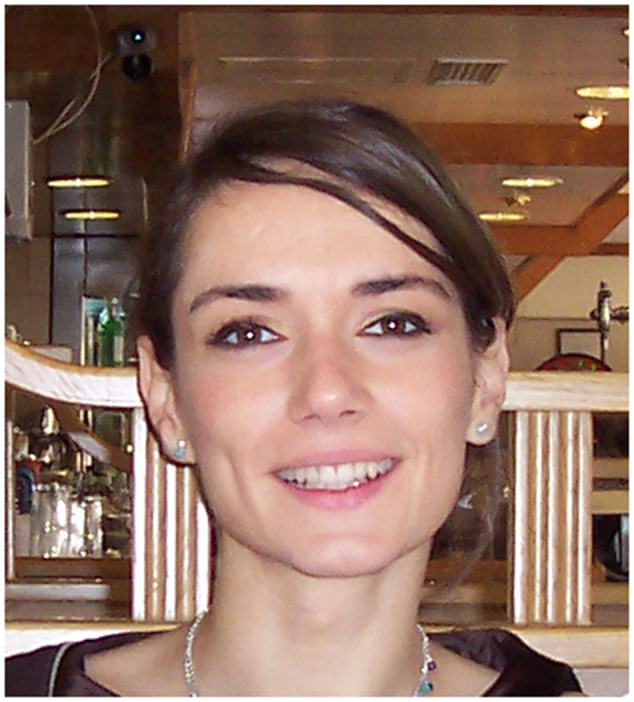
Laura Cif, provided by Laura Cif.

Laura Cif (Figure 8) is a neurologist born in 1975. She completed her specialist education in 2000 at the University of Montpellier, then pursued a master’s degree in neurosciences. In the following years, she continued her education at the University of Pierre and Marie Curie in Paris and at the University of Lille, then returned to Montpellier to complete her Ph.D. degree in 2011. Currently, she is an associate professor and consultant in movement disorders neurologist at the Unit of Functional Neurosurgery at the University Hospital Gui de Chauliac in Montpellier.

Her main research focus is on DBS in patients with various types of dystonia, especially on pediatric dystonia. DBS of the posteroventral internal globus pallidus (GPi) as a treatment for generalized dystonia was first introduced by Professor Philippe Coubes, a pediatric neurosurgeon in Montpellier (Coubes et al., 2000; Cif and Hariz, 2017). Joining his team, Dr. Cif analyzed the response to DBS in numerous subgroups of isolated and acquired dystonia (Castelnau et al., 2005; Cif et al., 2013, 2014; Cif and Coubes, 2017). The differences in response to DBS observed between individual cohorts contributed immensely to the indications and selection of patients for DBS, and to the various strategies in programming the stimulation, providing thus guidelines towards setting realistic expectations about the treatment.

### Neuropsychiatric Surgery

#### Gunvor Kullberg, Neurosurgeon

**Figure 9:**
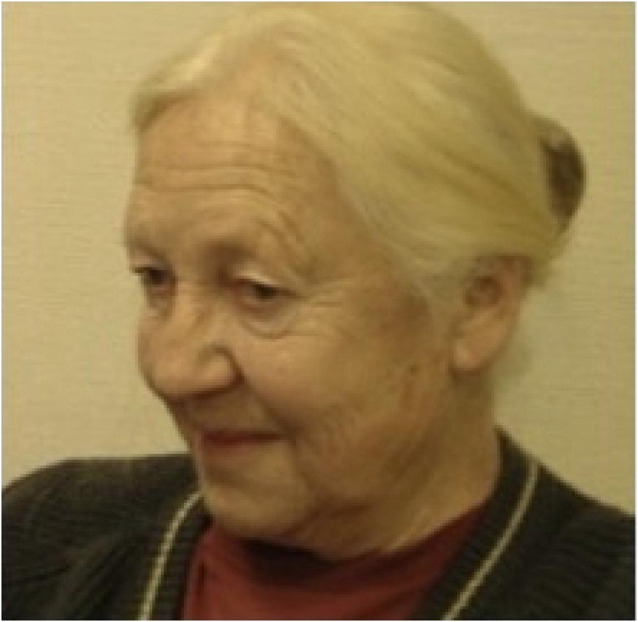
Gunvor Kullberg, reproduced from Hariz et al. (2014).

Gunvor Kullberg (Figure 9) is a Swedish neurosurgeon born in 1927. She started her career in 1955 as a psychiatrist at the University Hospital of Lund in Sweden. Two years later, an opportunity came to work at the Newcastle General Hospital in the UK. Here, after being exposed for the first time to the field of functional neurosurgery, she developed a significant interest in this discipline. As a consequence, she changed her specialization and completed her training as a neurosurgeon under the supervision of George Frederick Rowbotham. In 1960, she returned to the University Hospital of Lund where she conducted a wide array of research projects, involving stereotactic psychosurgery, the role of corticosteroids in postoperative brain edema, and studies using a then relatively new imaging method, the CT scan.

Using the 133Xe inhalation method she studied cerebral blood flow after stereotactic lesioning in psychiatric and Parkinson’s patients (Kullberg and Risberg, 1978, 1985). She demonstrated a decrease in cerebral blood flow in the prefrontal cortex following capsulotomy and frontobasal tractotomy and argued that this could indicate a lower prefrontal neural activity as a consequence of disruption in the neural pathways (Kullberg and Risberg, 1985). She also studied the evolution of stereotactic lesions using the CT scan and showed that corticosteroids could reduce the size of the surrounding edema (Kullberg et al., 1980; Cronqvist and Kullberg, 1983). Dr. Kullberg retired in the early 1990s and currently lives in Lund.

#### Helen S. Mayberg, Neurologist

**Figure 10:**
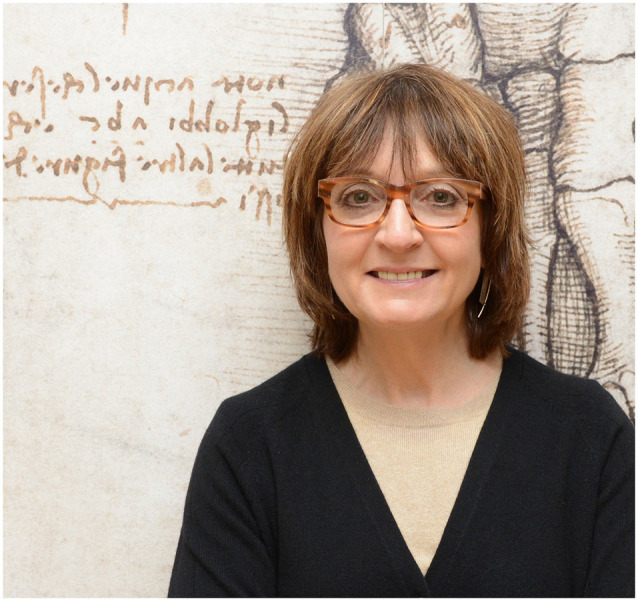
Helene Mayberg, provided by Helene Mayberg.

Helen S. Mayberg (Figure 10) is a neurologist born in 1956. She received a Bachelor of Arts degree in psychobiology from the University of California, Los Angeles at the age of 20 and a Medical Doctor degree 5 years later from the University of Southern California. After this, she obtained her certification as a neurologist from Columbia University in New York and a research fellowship in Nuclear Medicine at Johns Hopkins University. Among her multiple honors outstand her election as a member of the National Academy of Medicine of the United States of America, the American Academy of Arts and Sciences, and the National Academy of Inventors of the USA. Currently, she is Director and Professor at The Center of Advanced Circuit Therapeutics at the Mount Sinai Hospital in New York.

Her most influential work comprises the areas of functional neuroimaging and psychiatric disorders, in particular the pathophysiology of major depression. Prof. Mayberg has not only comprehensively examined the functional abnormalities of this disorder but also documented the mechanisms of antidepressant treatments in order to predict better clinical outcomes. These contributions led to the development of a novel target for DBS in patients with treatment-resistant depression, Brodmann area 25 also known as Cingulum 25 (Cg25), similar to the original target for subgenual cingulotomy performed by Lauri Laitinen between the 1950s and 1970s in Finland for various psychiatric disorders (Huotarinen et al., 2018). Together with Andres Lozano she pioneered DBS in the subgenual cingulate region in six patients with refractory depression (Mayberg et al., 2005). In this seminal study Mayberg and colleagues reported on changes in local cerebral blood flow in PET scans with reduced activity in the Cg25 and hypothalamus, as well as increased activity in prefrontal cortex and brainstem during DBS. These findings were equivalent to the metabolic changes observed after the administration of antidepressant medication (Mayberg et al., 2000). Four of the six patients in the pilot trial showed a significant reduction of the depressive symptomatology with unchanged medication after 6 months of DBS. Since then, several studies have indicated that Cg25 DBS is an effective and safe treatment for patients with treatment-resistant depression (Puigdemont et al., 2015; Merkl et al., 2018). Nevertheless, this therapy still remains experimental pending enrollment of larger samples of patients. Prof. Mayberg continues her multidisciplinary research in the fields of neurology, psychiatry, neurosurgery, imaging, and neuroscience as acting Director of The Nash Family Center for Advanced Circuit Therapeutics at the Icahn School of Medicine at Mount Sinai.

#### Veerle Visser-Vandewalle, Neurosurgeon

**Figure 11:**
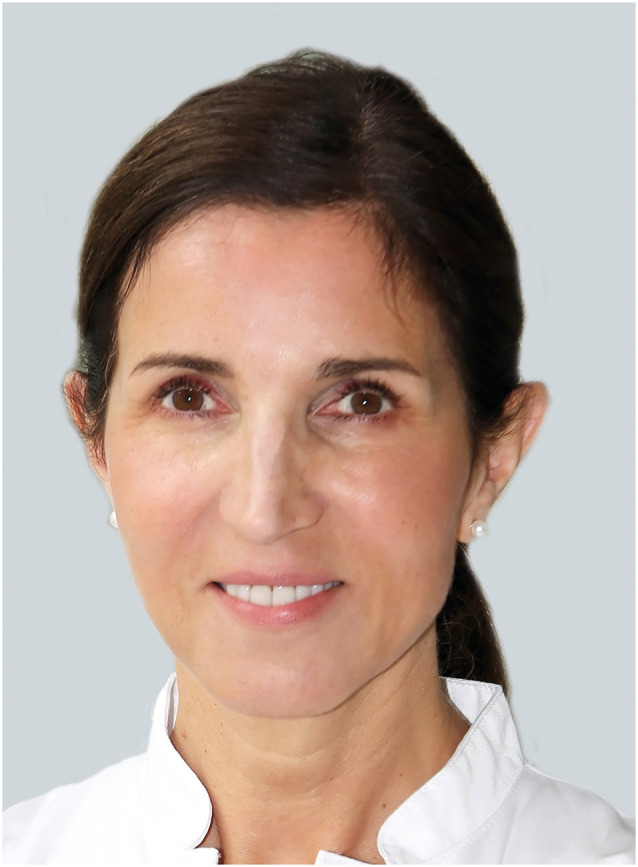
Veerle Visser-Vandewalle, provided by Veerle Visser-Vandewalle.

Veerle Visser-Vandewalle (Figure 11) is a neurosurgeon born in 1964. She completed her training in neurosurgery at the AZ St-Jans Hospital, in Bruges and the University Hospital of Ghent in Belgium between 1989 and 1996, where she performed her first DBS procedures in movement disorders.

In 1999, DBS was introduced in the field of neuropsychiatric disorders by Dr. Visser-Vandewalle and colleagues in Ghent, Belgium (Vandewalle et al., 1999). In this pioneering work, a 42-year old patient with intractable Gilles de la Tourette syndrome (GTS) received thalamic DBS electrodes spanning the centromedian nucleus, the nucleus ventro oralis internus and the substantia periventricular. The targeting procedure was based on the thalamic stereotactic lesions in GTS patients performed by Hassler and Dieckmann in the 70s (Hassler and Dieckmann, 1970). Postoperatively, the patient showed a significant reduction of tics, as well as a decrease in comorbid self-injurious behavior. Since then, numerous studies have replicated these positive findings in GTS patients after stimulation of thalamic targets and of other subcortical areas.

She continued her work in DBS and functional neurosurgery at the Maastricht University Medical Center in the Netherlands for 13 years. During this time, she obtained a Ph.D. degree with a dissertation about her work in DBS and GTS. She was also named full professor in Functional Neurosurgery in 2007. In 2012, she was appointed as Head of the Department of Stereotactic and Functional Neurosurgery at the University Hospital of Cologne in Germany, where she currently holds this position.

### Neurophysiology

#### Denise Albe-Fessard (1916*–2003†), Neurophysiologist

**Figure 12:**
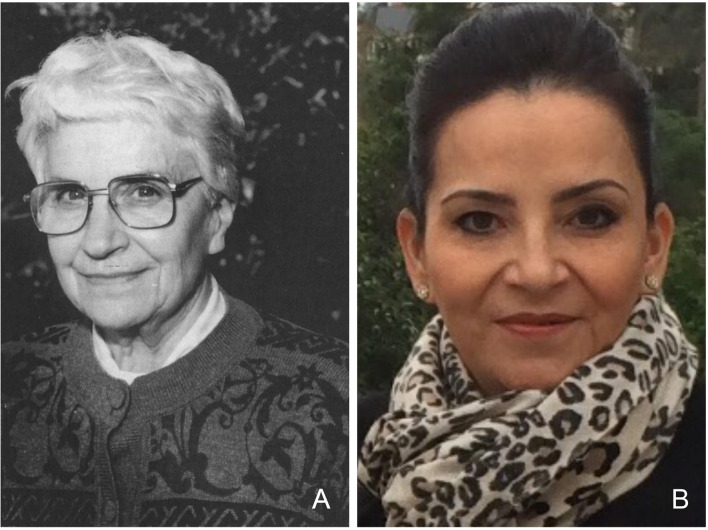
**(A)** Denise Albe-Fessard, reproduced from Hariz et al. (2014). **(B)** Ana Luisa Velasco, provided by Ana Luisa Velasco.

Densise Albe-Fessard (Figure 12A) was a neurophysiologist born in 1916. She graduated from the School of Physics and Chemistry in Paris with an Engineering degree in 1937 (Albe-Fessard, 1998). In 1950 she obtained a Ph.D. degree from the University of Paris. Her first experiments in the field of neurophysiology were performed in Paris and then shortly in Bordeaux during the Second World War.

The use of intracerebral microrecordings is nowadays a worldwide common practice during functional stereotactic procedures. This technique provides precise information about the position of the electrode and ultimately allows to differentiate between different subcortical structures. The pioneering work that led to the development of intraoperative microrecording as we know it was performed by Denise Albe-Fessard.

After the Second World War, she continued her work at the French National Centre for Scientific Research (CNRS) where she studied neurophysiology in electric fish. After learning and perfecting the method to make glass-electrodes, she applied this technique to the large cells of the cat somatomotor cortex. She continued her work in primates after being appointed as faculty at the Sorbonne University Pierre and Marie Curie in Paris in the 1950s, where she mainly focused on the thalamic pathways. These insights allowed her to perform the first microrecordings in humans in the beginning of the 1960s in collaboration with the French neurosurgeon Gerard Guiot at the Hospital Foch in Suresnes near Paris. During these procedures, she documented the presence of rhythmic thalamic activity that was associated with the tremor frequency (Guiot et al., 1962; Albe-Fessard et al., 1967), and she advocated intraoperative stimulation at high frequency to stop the tremor. Her work extended to the delineation of several subcortical networks, in particular, movement disorders and pain pathways through thalamic pain processing. These achievements led her to be elected the first president of the International Association for the Study of Pain (IASP) in 1975. She was the first woman to receive the Spiegel-Wycis Award in 1989 and to be awarded the distinction as Knight of the Legion of Honor and Officer of the Order of Merit by the French government. Her vast legacy in the field of neurophysiology has been continued by her multiple students and colleagues worldwide that currently lead the field of modern neuromodulation.

#### Ana Luisa Velasco, Neurologist

Ana Luisa Velasco (Figure 12B) is a Mexican neurologist and neurophysiologist born in 1962. She obtained a Medical Doctor degree from the National Autonomous University of Mexico (UNAM) in 1988. After this, she completed her Neurology Training at Mexico General Hospital in 1991. During this time, she started working in the field of neurophysiology and epilepsy and developed a personal interest in these areas.

Her board certification thesis focused on EEG and MRI abnormalities in children with Lennox-Gastaut syndrome and epilepsia partialis continua (Velasco et al., 1993). At this early point of her career, she started working with two of her most important mentors, Marcos Velasco and Francisco Velasco. The former, her father, was himself a neurophysiologist specialized in the study of epilepsy, and the latter, her uncle, a neurosurgeon dedicated to the field of functional neurosurgery. From this singular combination of expertise, she worked with them on electrical stimulation of the thalamus to treat patients with severe refractory seizures. Eventually, she received the prestigious Fogarty International Research Fellowship and spent 2 years at the Brain Research Institute at the University of California, Los Angeles under the tutorship of Jerome Engel Jr. and Charles Wilson. Here, she had the opportunity to study epilepsy from different angles including neuropsychology, neuroimaging, clinical, surgical, and research techniques. Following her return to Mexico, she obtained a Ph.D. degree in biomedical sciences and obtained her certification as a Neurophysiologist. At this point, she founded the Epilepsy Clinic at the Mexico General Hospital, where she worked as Head of the Clinic. During the coming years, she devoted herself to the study of the modulation of the brain through electrical stimulation of different cerebral targets to control refractory seizures. This resulted in diverse seminal publications about the chronic stimulation of the hippocampus and the centromedian thalamic nuclei in the treatment of epilepsy (Velasco et al., 2006, 2007a,b). Among her multiple honors are her election as a member of the Mexican Academy of Surgery in 2010 and the Mexican National Academy of Medicine in 2013. In 2019, she was appointed Head of the Department of Neurology at the Mexico General Hospital in Mexico City, where she still holds this position and continues her clinical and academic activities in the field of neuromodulation.

### Neurotechnology

#### Zelma Kiss, Neurosurgeon

**Figure 13:**
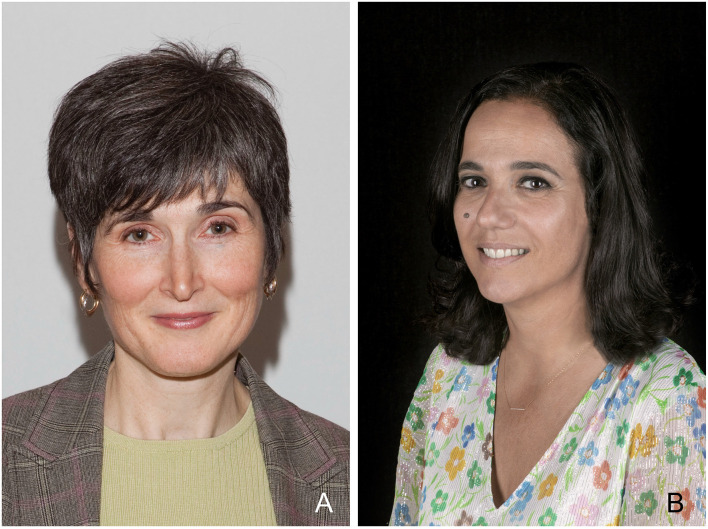
**(A)** Zelma Kiss, provided by Zelma Kiss. **(B)** Jocelyn Bloch, provided by Jocelyn Bloch.

Zelma Kiss (Figure 13A) is a neurosurgeon born in 1964. She graduated from the medical faculty of the University of Ottawa, Canada at the age of 24. She completed her neurosurgical training at the University of Toronto, where she also received her Ph.D. After winning the Van Wagenen fellowship, she had the opportunity to continue her postdoctoral education under the supervision of Alim Louis Benabid in Grenoble, France. In 2000, she was appointed at the University of Calgary, where she is currently an associate professor of the Department of Clinical Neurosciences and is the Head of the Neuromodulation program of southern Alberta.

The main research interest of Dr. Kiss is the therapeutic mechanism of DBS in different brain areas. Using rodent models, she has thoroughly analyzed the membrane depolarization after thalamic stimulation, concluding that DBS operates through disruption of the rhythmic firing of the tremor cells (Kiss et al., 2002). Likewise, she has been largely involved in the study of the mechanism of action during globus pallidus internus DBS. Particularly, on the question of why pallidal DBS shows an almost immediate effect in Parkinson’s patients, while for dystonia it can take weeks or months of stimulation for patients to profit fully from chronic stimulation (Hristova et al., 2000; Coubes et al., 2004). In this respect, she has studied the entopenduncular nucleus in rats during stimulation, a representative for the human globus pallidus, while recording local field potentials in the motor cortex, the striatum, and the ventroanterior thalamus (McCracken and Kiss, 2014). The results from these experiments showed that local field potentials changed over time under continuous DBS, demonstrating how the therapeutic effects of DBS could gradually develop differently after its chronic usage. Her research has immensely contributed to understanding the basic principles of DBS and how this therapy is applied in humans. As a co-editor to “Brain Stimulation” she was the first editor to publish recently an editorial criticizing the lower battery capacity found in new versions of DBS hardware and she drew attention to the need to report this to government approval bodies (Kiss and Hariz, 2019).

#### Jocelyn Bloch, Neurosurgeon

Jocelyn Bloch (Figure 13B) is the Head of the Stereotactic and Functional Neurosurgery team at the University Hospital in Lausanne, Switzerland. She graduated from the medical faculty at the University of Lausanne in 1994 and completed her neurosurgical training in Lausanne and Zurich.

After this, she specialized in functional neurosurgery. From 1997 to 1999, she joined the team of Prof. Patrick Aebischer where she studied gene therapy and translational sciences, which contributed considerably to her later projects. She is vice-president of the European Society for Stereotactic and Functional Neurosurgery.

Together with Jean-Françoise Brunet, she studied the effect of autologous transplantation of adult brain cells in primates after injury to the motor cortex, as well as in monkeys treated with MPTP, modeling Parkinson’s disease (Kaeser et al., 2011; Bloch et al., 2014). The promising results obtained from these studies offer a good alternative to other sources of neural transplantation, such as fetal stem cells or genetically modified pluripotent adult somatic cells.

Dr. Bloch has also been actively involved in the field of neuroprosthetics in patients with spinal cord injury. Using microelectrode arrays in the motor cortex and epidural electrical stimulation of the lumbar and the cervical spine, her team conducted ground-breaking research on this topic first in primates (Capogrosse et al., 2016; Barra et al., 2018). Based on the spatiotemporal pattern of motor neuron activation in the lumbar spine during movements and the matching cortical activity in the motor area, they developed a wireless system with a decoder that creates a neural bridge between both areas. After sufficient calibration of the system, they inflicted a lesion in the corticospinal tracts. With the help of brain-controlled sub-lesion stimulation, the monkeys could regain their walking abilities shortly after the surgery (Capogrosse et al., 2016) Based on these studies, in 2018 they developed a wireless, voice-controlled lumbar epidural stimulation system for patients with spinal cord injury (Wagner et al., 2018). With the help of epidural electrical stimulation and months of rehabilitation with the implant, they could significantly improve the patients’ ability to walk and stand, and they even showed improvement off stimulation. The innovative work of Dr. Bloch raises hope for patients with spinal cord injury and provides a basis for new applications of functional neurosurgery.

### Neuroimaging

#### Mojgan Hodaie, Neurosurgeon

**Figure 14:**
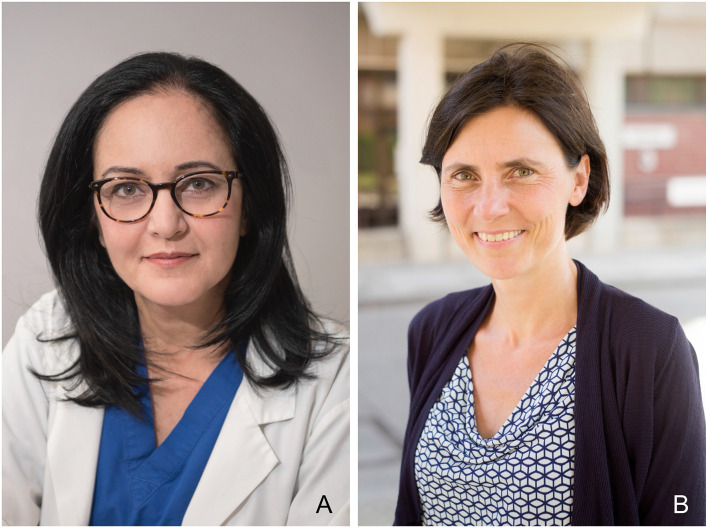
**(A)** Mojgan Hodaie, provided by Mojgan Hodaie. **(B)** Andrea Kühn, provided by Andrea Kühn.

Mojgan Hodaie (Figure 14A) is a neurosurgeon at the Toronto Western Hospital and Professor at the Department of Surgery at the University of Toronto and Institute of Medical Sciences in Canada. She obtained her Medical Doctor degree from Queen’s University at Kingston, Canada in 1996. After this, Mojgan Hodaie completed her Neurosurgery Training in 2003 and a Fellowship in Stereotactic and Functional Neurosurgery in 2004 at the University of Toronto. As an established neurosurgeon, she focused on the management of several functional neurological disorders, particularly in the surgical treatment of trigeminal neuralgia and facial pain (Hodaie et al., 2012). Her contributions to this field combine neuroimaging and radiosurgery. She has investigated brain abnormalities in sensory and motor areas using tractography to visualize these microstructural neuroanatomical changes (Hodaie et al., 2010; Desouza et al., 2013). Hodaie and colleagues have observed gray matter abnormalities in patients with trigeminal neuralgia in anatomical structures involved in pain perception and modulation. These morphometric analyses have shown increased volumes in amygdala, sensory nuclei of the thalamus, periaqueductal gray and basal ganglia, as well as higher cortical thickness in the primary somatosensory cortex and frontal lobes in patients with trigeminal neuralgia when compared to healthy subjects (Desouza et al., 2013). These methods could be used to identify biomarkers in pain syndromes and eventually help to adjust therapies or indicate surgical procedures.

Mojgan Hodaie has devoted herself to distance teaching of functional neurosurgery especially in developing countries (Blankstein et al., 2011). She founded the NEURON (Neurosurgical Education with Universal Reach Online) project, which has implemented online courses in the field of neurosurgery around the world, and she is a member of the Foundation of International Education in Neurological Surgery (FIENS). Her contributions to the academic and scientific fields have resulted in multiple honors and awards, including the Grand Cross of the Legion of Honor of Monisaraphon by the Kingdom of Cambodia. She was also elected as an officer of the Board of Directors of the World Society for Stereotactic and Functional Neurosurgery (WSSFN).

#### Andrea Kühn, Neurologist

Andrea Kühn (Figure 14B) is a neurologist and neuroscientist born in 1970. She studied medicine at the Charité and at the Henri Poincaré University in Nancy, France. After obtaining her medical degree, she worked as a postdoctoral researcher at University College of London under the mentorship of Peter Brown from 2002 till 2007, where her main focus was studying local field potentials in the STN in Parkinson’s patients (Doyle et al., 2005; Kühn et al., 2005, 2006; Williams et al., 2005).

In 2010, upon her return to Berlin, she worked as a neurologist at the UniversityHospital Charité where she became head of the Movement Disorders and Neuromodulation Unit. Her primary research interest became pathological oscillatory activity in patients with movement disorders under deep brain stimulation (Barrow et al., 2014; Neumann et al., 2017, 2019), which later enabled the development of closed loop stimulation. She also accomplished groundbreaking work in the field of neuroimaging. Together with Andreas Horn, she presented “Lead-DBS” in 2014, an open-access toolbox for localization and visualization of DBS electrodes (Horn and Kühn, 2015). With the help of postoperative MRI and CT imaging, this tool allows semiautomated reconstruction of the DBS electrodes and the position of their contacts within the targeted regions. This software can assist clinicians and researchers to generate a virtual reconstruction of the stimulated structures (Horn et al., 2017).

### Conclusion

**Figure 15:**
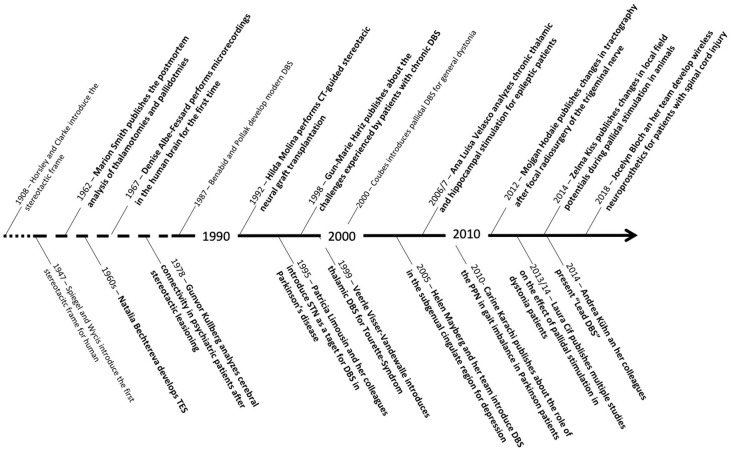
Timeline showing some of the most important scientific contributions of the women mentioned in this review in relation to other major scientific advances in the field of stereotactic and functional neurosurgery.

In this review, we summarized the professional achievements of 16 exceptional women working in the field of neuromodulation and functional neurosurgery. Although they are from different backgrounds, including neuropathology, neurophysiology, neurology, and neurosurgery, they all contributed to the development and evolution of stereotactic and functional neurosurgery as we know it today. Fortunately, this summary shows that in the last two decades an increasing number of women have joined these disciplines and many of them are currently in high-ranking academic positions (Figure 15). Without a doubt, their scientific and medical contributions will inspire future generations of researchers, both women and men, to participate in the development of stereotactic and functional neurosurgery.

## Author Contributions

Conceptualization, visualization, and investigation: PH and PA. Methods, writing—original draft, and writing—review and editing: PH, JP, and PA. All authors contributed to the article and approved the submitted version.

## Conflict of Interest

The authors declare that the research was conducted in the absence of any commercial or financial relationships that could be construed as a potential conflict of interest.

## Publisher’s Note

All claims expressed in this article are solely those of the authors and do not necessarily represent those of their affiliated organizations, or those of the publisher, the editors and the reviewers. Any product that may be evaluated in this article, or claim that may be made by its manufacturer, is not guaranteed or endorsed by the publisher.
