# Corrigendum: Women in Neuromodulation: Innovative Contributions to Stereotactic and Functional Neurosurgery

**DOI:** 10.3389/fnhum.2022.859587

**Published:** 2022-03-17

**Authors:** Petra Heiden, Julia Pieczewski, Pablo Andrade

**Affiliations:** ^1^Department of Neurosurgery, Faculty of Medicine, University of Cologne, Cologne, Germany; ^2^Department of Stereotactic and Functional Neurosurgery, Faculty of Medicine, University of Cologne, Cologne, Germany

**Keywords:** functional neurosurgery, neuromodulation, stereotactic surgery, deep brain stimulation, history, women

In the original article, there was errors regarding the biographical data of “Helen S. Mayberg, Zelma Kiss and Andrea Kühn.”

A correction has been made to the section **Neuropsychiatric Surgery**, **Helen S. Mayberg, Neurologist**, paragraph 1:

Helen S. Mayberg (Figure 10) is a neurologist born in 1956. She received a Bachelor of Arts degree in psychobiology from the University of California, Los Angeles at the age of 20 and a Medical Doctor degree 5 years later from the University of Southern California. After this, she obtained her certification as a neurologist from Columbia University in New York and a research fellowship in Nuclear Medicine at Johns Hopkins University. Among her multiple honors outstand her election as a member of the National Academy of Medicine of the United States of America, the American Academy of Arts and Sciences, and the National Academy of Inventors of the USA. Currently, she is Director and Professor at The Center of Advanced Circuit Therapeutics at the Mount Sinai Hospital in New York.

A correction has been made to the section **Neurotechnology**, **Zelma Kiss, Neurosurgeon**, paragraph 1:

Zelma Kiss (Figure 13A) is a neurosurgeon born in 1964. She graduated from the medical faculty of the University of Ottawa, Canada at the age of 24. She completed her neurosurgical training at the University of Toronto, where she also received her Ph.D. After winning the Van Wagenen fellowship, she had the opportunity to continue her postdoctoral education under the supervision of Alim Louis Benabid in Grenoble, France. In 2000, she was appointed at the University of Calgary, where she is currently an associate professor of the Department of Clinical Neurosciences and is the Head of the Neuromodulation program of southern Alberta.

A correction has been made to the section **Neuroimaging**, **Andrea Kühn, Neurologist**, paragraph 2:

In 2010, upon her return to Berlin, she worked as a neurologist at the University Hospital Charité where she became head of the Movement Disorders and Neuromodulation Unit. Her primary research interest became pathological oscillatory activity in patients with movement disorders under deep brain stimulation (Barrow et al., 2014; Neumann et al., 2017, 2019), which later enabled the development of closed loop stimulation. She also accomplished groundbreaking work in the field of neuroimaging. Together with Andreas Horn, she presented “Lead-DBS” in 2014, an open-access toolbox for localization and visualization of DBS electrodes (Horn and Kühn, 2015). With the help of postoperative MRI and CT imaging, this tool allows semiautomated reconstruction of the DBS electrodes and the position of their contacts within the targeted regions. This software can assist clinicians and researchers to generate a virtual reconstruction of the stimulated structures (Horn et al., 2017).

The authors apologize for this error and state that this does not change the scientific conclusions of the article in any way. The original article has been updated.

## Publisher's Note

All claims expressed in this article are solely those of the authors and do not necessarily represent those of their affiliated organizations, or those of the publisher, the editors and the reviewers. Any product that may be evaluated in this article, or claim that may be made by its manufacturer, is not guaranteed or endorsed by the publisher.
